# Chemical composition, nutrient-balancing and biological treatment of hand washing greywater

**DOI:** 10.1016/j.watres.2018.07.005

**Published:** 2018-11-01

**Authors:** Christopher Ziemba, Odile Larivé, Eva Reynaert, Eberhard Morgenroth

**Affiliations:** a Eawag: Swiss Federal Institute of Aquatic Science and Technology, 8600 Dübendorf, Switzerland; b ETH Zürich, Institute of Environmental Engineering, 8093 Zürich, Switzerland; c EPFL Lausanne, Environmental Chemistry Laboratory, 1015 Lausanne, Switzerland

**Keywords:** Soap, Nitrogen, Micro-nutrients, Biologically activated membrane bioreactor (BAMBi), Gravity-driven membrane (GDM), Handwashing

## Abstract

On-site biological hand washing water treatment can improve global access to safe hand washing water, but requires a thorough understanding of the chemical composition of the water to be treated, and an effective treatment strategy. This study first presents a detailed characterization of the individual inputs to hand washing water. We demonstrate (i) that soap is likely the most significant input in hand washing water, representing ∼90% of mass loading, and (ii) that inputs to hand washing water have low concentrations of biologically-essential macro- and micro-nutrients (nitrogen, phosphorus, potassium, copper, zinc, molybdenum and cobalt) with respect to carbon, which may impair biological carbon removal. This study next formulates a recipe that recreates a representative composition of hand washing water and develops a procedure to identify and supplement nutrients in which this recipe is estimated to be deficient. Batch testing of the nutrient-supplemented hand washing water with an inoculum of planktonic bacteria demonstrated improved assimilable organic carbon removal (99% vs. 86% removal) and produced lower final dissolved organic carbon concentrations (1.7 mg_C_/L vs. 3.5 mg_C_/L) compared to realistic (nutrient-deficient) washing water. Supplementing nutrients did promote cell growth (50x higher final total cell count). Full-scale testing in a biologically activated membrane bioreactor (BAMBi) system treating 75 L/day of nutrient-supplemented hand washing water showed that long-term operation (100 days) can deliver effective carbon removal (95%) without detrimental fouling or other disruptions caused by cell growth. This work demonstrates that biological treatment in a BAMBi system, operated with appropriate nutrient-balancing offers an effective solution for decentralized treatment of light greywater.

## Highlights

• Soap is the dominant ingredient in hand washing water, accounting for 90% dry mass.
• Hand washing water is generally biologically nutrient-deficient, relative to carbon.
• Supplementing nutrients into hand washing water can improve carbon removal.
• Supplementing nutrients can also promote cell growth.
• 95% carbon removal was achieved in a full-scale biological treatment system.

## Graphical abstract

**Figure:**
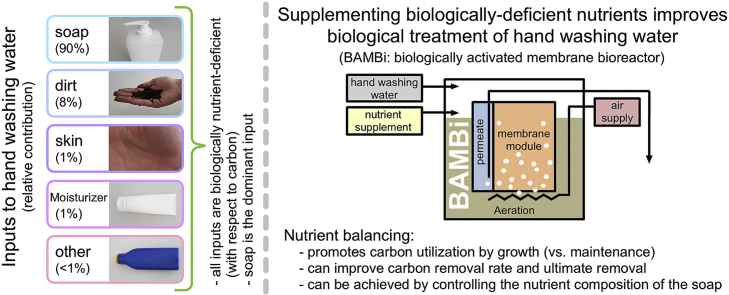

## 1 Introduction

Biological treatment processes are essential to meeting current discharge-oriented wastewater treatment objectives, and we must also look to biological processes to provide water recycling capability to meet the expanding water demands of the future. The wastewater stream that can most easily be recovered and recycled for high-quality water demands is greywater, and more specifically light greywater derived from hand washing and showering, due primarily to reduced organic and nutrient loading compared to other wastewater types (Eriksson et al., 2002). Effective biological treatment of greywater, or any other wastewater, requires a balance between biologically-essential nutrients. The microbial communities that perform the treatment require a variety of essential nutrients for growth and maintenance functions, and the exhaustion of an essential nutrient may limit the removal of other nutrients from the wastewater (Grady et al., 2011). Effective biological treatment of wastewaters that are not initially nutrient-balanced can be achieved by supplementing the deficient nutrients (Jefferson et al., 2001; LeChevallier et al., 1991).

Previous studies have estimated nutrient requirements and demonstrated improvements in carbon removal during wastewater treatment following nutrient supplementation (Burgess et al., 1999; Jefferson et al., 2001). Other studies have demonstrated effective biological treatment of greywater without any nutrient supplementation (Gross et al., 2007). Whether or not supplementing nutrients improves treatment will depend on the specific source and nutrient composition of the water to be treated. While tremendous variations have been observed in greywater samples collected in different parts of the world (Al-Jayyousi, 2003; Friedler, 2004; Ghaitidak and Yadav, 2013), part of the variation is based on which inputs are included in the broad category of greywater, such as kitchen sink and laundry sources. Light greywater inputs, such as bath, shower or hand washing, generally contain significantly less dissolved organic carbon, nitrogen and phosphorus than dark greywater inputs such as kitchen sinks, dishwashers or washing machines (Friedler, 2004). Therefore, understanding the relative volume contributions of specific inputs, and the nutrient composition of each specific input, impacts design choices for treatment and reuse strategies, specifically with respect to meeting nutrient requirements for biological systems. No existing study has systematically investigated the quantities and composition of different material inputs to greywater, analyzing both the biological compatibility of the carbon and the concentrations provided of other biologically-essential nutrients.

### 1.1 Understanding the inputs and composition of hand washing water

The composition of any wastewater is the sum of materials in the initial water and all the materials that are added during usage. In the case of hand washing water, the additional materials consist of soap, whatever dirt or undesirable materials are intentionally washed off the hands, but also traces of personal care products and skin cells. Existing literature can contribute to our understanding of hand washing water inputs by two different approaches. Existing literature investigating greywater or greywater treatment often present chemical characterization of real-world hand washing water. These studies generally do not investigate the quantity or composition of contributing sources or fully analyze the influent water, though they can provide information about what the individual contributions can add up to. The organic carbon (OC) is generally expressed as total organic carbon (TOC) or dissolved organic carbon (DOC), but not biologically-compatible assimilable organic carbon (AOC). The second tool we have in existing literature to understand hand washing water inputs is the collection of recipes for synthetic greywater that have been designed, in part, to recreate the chemical and physical characteristics of measured greywater, often with some (though incomplete) consideration for individual inputs.

#### 1.1.1 Measurements from real-world hand washing water

While numerous studies have measured the chemical and physical characteristics of combined greywater, measurements specifically for hand washing water are scarce. Results from studies that have reported measurements from hand washing sinks are summarized in Table 1. Additional information on each study is presented in Supporting Information Section S.1.

**Table 1:** Composition of real hand washing water (HWW) found in literature.

References	Laak (1974)	Surendran and Wheatley (1998)	Almeida et al. (1999)	Al-Jayyousi (2003)	Jefferson et al. (2004)	Friedler (2004)	Jamrah et al. (2008)
**Origins of HWW**	five households in the USA	university residence halls in the UK	two households in the UK	not specified	102 individuals in the UK	Israel	Oman
**Oxygen demand** **(mg_O2_/L)**							
Biochemical (BOD)	236	252		109 (5 days)	155 (5 days)	205 (total)	100 (5 days)
						93 (dissolved)	
Chemical (COD)	383	433	298 (total)	263	587		110
			221 (dissolved)				
**Chemical composition** **(mg_C_/L, mg_N_/L, mg_P_/L)**							
Total organic carbon (TOC)		40			99	119 (total)	63
						74 (dissolved)	
Nitrogen (N)	1.15 (NH_4_^+^)	0.53 (NH_4_^+^)	0.3 (NH_4_^+^)	9.6 (total)	10.4 (total)	0.39 (NO_3_^−^)	10.2 (NO_3_^−^)
	0.28 (NO_3_^−^)	0.34 (NO_3_^−^)	6 (NO_3_^−^)			0.39 (NH_4_^+^)	
Phosphorus (P)	48.8	45.5	13.3	2.58	0.13	15	
**Nutrient ratios** **(mg_N_/mg_C_, mg_P_/mg_C_)**							
N/TOC		0.02			0.11	0.007	0.16
P/TOC		1.13			0.001	0.13	

The reported BOD of hand washing water varies between 100 and 252 mg_O2_/L and the COD between 110 and 587 mg_O2_/L. As the concentrations of ammonium and other non-carbon oxidizable constituents are low in hand washing water (between 0.39 mg _NH4-N_/L and 1.15 mg_NH4-N_/L) we assume that BOD and COD values are dominated by the oxidation of the carbon. The BOD/COD ratio can thus also be used to estimate the biodegradability of the carbon. The BOD/COD ratios for the studies presented in Table 1 range from 0.3 to 0.9, but average 0.6, which is 50% higher than the ratio of 0.4 reported for typical domestic wastewaters (Tchobanoglous et al., 2014). Total nitrogen (TN)/TOC ratios for measured hand washing water range between 0.007 and 0.16 mg_N_/mg_C_, which is lower than the TN/TOC ratio of 0.21 mg_N_/mg_C_ for typical domestic wastewaters (Tchobanoglous et al., 2014). High phosphorus concentrations (up to 48.8 mg_P_/L) can potentially be explained by the use of phosphorus-based detergents. Phosphate-based detergents are currently banned in many regions, including the European Union and the United States, indicating that only the concentrations reported by Jefferson et al. (2004) and Al-Jayyousi (2003) should be used as references. The phosphorus (P)/TOC ratio from Jefferson et al. (2004) is only 0.001 mg_P_/mg_C_, which is much lower than the P/TOC ratio of 0.03 mg_P_/mg_C_ for a typical domestic wastewater (Tchobanoglous et al., 2014).

To this date, only little data are available on micro-nutrients in hand washing water. Jamrah et al. (2008) reported concentrations of potassium, zinc, lead, copper and nickel. Friedler (2004) analyzed samples for silver, chromium, nickel, cadmium, manganese, copper, lead and zinc, but found most concentrations to be below the detectable limit. We have not identified any study that reports concentrations for all the nutrients regarded as being always or mostly essential for biological treatment (Egli, 2009). Even the two studies (Friedler, 2004; Jamrah et al., 2008) which provide the most detailed micro-nutrient analysis do not differentiate the nutrients contributed during hand washing, from the nutrients already contained in the water before washing. What we can say is that we expect greywater may be more easily biodegradable than municipal wastewater, but contains lower nutrient to carbon ratios for essential nutrients including nitrogen and phosphorus.

#### 1.1.2 Synthetic artificial greywater and hand washing water

Laboratory testing of greywater treatment systems often utilizes synthetic wastewater to provide a consistent feed material (Diaper et al., 2008; Hourlier et al., 2010). While no synthetic greywater recipe is known specifically for hand washing water, many synthetic greywater recipes exist for combined greywater, or greywater from unspecified inputs. Table 2 presents several recipes. The main inputs considered in these greywater recipes are detergents (soap, shampoo and laundry), personal care products, organic matter (e.g., from dirt, kitchen sinks and human body), inorganic matter (e.g., from dirt) and microbial contamination. Some of the recipes also include buffers to maintain a stable pH and some salts to mimic a realistic composition of real greywater.

**Table 2:** Types and quantities of compounds used in synthetic greywater recipes found in literature. GW: greywater.

Source	Hourlier et al. (2010)	Diaper et al. (2008)	Nghiem et al. (2006)	Jefferson et al. (2001)	Surendran and Wheatley (1998)
Type of GW	bathroom	laundry and bathroom	not defined	not precise, real greywater in the UK	combined greywater (including kitchen sink)
**Soap, shampoo and laundry**					
	Sodium dodecyl sulfate: 50 mg/L	Shampoo/hand wash: 720 mg/L		Synthetic soap: 64 mg/L	Shampoo: 0.1 mL/L
	Glycerol: 200 mg/L	Laundry: 150 mg/L		Hair shampoo: 0.6 mL/L	Washing powder: 30 mg/L
**Personal care products**					
	Glycerol (included above)	Sunscreen or moisturizer: 15 or 10 mg/L respectively			
		Toothpaste: 32.5 mg/L			
		Deodorant: 10 mg/L			
**Organic matter (e.g. from dirt, kitchen sinks and human body)**					
	Cellulose: 100 mg/L	Lactic acid: 28 mg/L	Cellulose: 50 mg/L	Sunflower oil: 0.01 mL/L	Dextrin: 85 mg/L
	Lactic acid: 100 mg/L	Boric acid: 1.4 mg/L	Humic acid: 20 mg/L		Soluble starch: 55 mg/L
		Vegetable oil: 7 mg/L	Sunflower oil: 0.01 mL/L		Yeast extract: 50 mg/L
					Cooking oil: 0.1 mL/L
**Inorganic matter (e.g. from dirt)**					
		Kaolin clay: 50 mg/L	Kaolin clay: 50 mg/L		
**Microbial contamination**					
	Septic effluent: 10 mg/L	Secondary effluent: 2 mL/L		Tertiary effluent: 2.4 mL/L	Settled sewage: 10 mL/L
**Buffer**					
	Sodium hydrogen carbonate: 70 mg/L	Sodium hydrogen carbonate: 25 mg/L	Sodium hydrogen carbonate: 85 mg/L		Sodium hydrogen carbonate: 55 mg/L
**Salts**					
	Sodium sulfate: 50 mg/L	Sodium sulfate: 35 mg/L	Sodium chloride: 10 mM		Ammonium chloride:75 mg/L
		Disodium phoshphate: 39 mg/L	Calcium chloride: 0.5 mM		Sodium dihydrogen phosphate: 11.5 mg/L
					Potassium sulfate: 4.5 mg/L

Most greywater recipes use commercial soaps, shampoos and detergents to recreate these contributions (Diaper et al., 2008; Jefferson et al., 2001; Surendran and Wheatley, 1998). Hourlier et al. (2010), however, used sodium dodecyl sulfate (SDS) and glycerol to recreate the contributions of soap and shampoo. Few synthetic greywater recipes account for the contributions of personal care products. Diaper et al. (2008) used commercially-available sunscreen, moisturizer, toothpaste and deodorant and Hourlier et al. (2010) added glycerol to represent moisturizing agents. Organics are added in various forms like cellulose, lactic acid, humic acid or oil. Two greywater recipes also add inorganic matter in the form of kaolin clay (Diaper et al., 2008; Nghiem et al., 2006). Many recipes add sodium hydrogen carbonate as a natural buffer, as it is usually also present in tap water (Diaper et al., 2008; Hourlier et al., 2010; Jefferson et al., 2001; Surendran and Wheatley, 1998). Sodium, calcium, phosphate and ammonium have also been added separately to recreate concentrations in measured greywater.

Future work with synthetic greywater requires understanding how existing recipes can be improved, but also what they do well. The main drawbacks of existing recipes are a general lack of information regarding (i) the specific type of greywater (heavy vs light) and specific inputs (kitchen sink or no, with in-sink grinder or no) the recipe intents to mimic, (ii) a complete understanding of how the amounts and types of ingredients used in the synthetic recipes were selected, (iii) the exact composition of the added ingredients, and (iv) a lack of attention to the quantities and the availabilities of biologically-relevant nutrients. A generic or a non-specific synthetic recipe for greywater that may mimic kitchen waste, will not as accurately reflect realistic performance in a treatment system as would a recipe designed specifically for hand washing water. Some recipes have taken steps to link ingredients to inputs (such as hand soap, skin, organic and inorganic dirt and personal care products), but not in a complete or quantifiable fashion. Being complete and quantifiable is necessary to more accurately create a synthetic recipe to reflect a specific input. Once we build an understanding of inputs, we must build a recipe out of reproducible reagents. As many existing synthetic greywater recipes use commercial products, with compositions that can vary depending on the geographic region and over time, they cannot be reproduced accurately. The importance of reproducible ingredients has also been stressed in the work of Abed and Scholz (2016), however their recipe also relies on secondary treatment effluent to supply micronutrients. The work of Jefferson et al. (2001) has indeed noted the importance of nutrients in greywater treatment, and this attention must continue. Additional information for each recipe in the table, and the work of Abed and Scholz (2016) is presented in Supporting Information Section S.2.

### 1.2 Nutrient requirements for biological treatment

One approach to estimate nutrient requirement for a biological system is based on the elemental composition of the microbial cells (Egli, 2009). Harvested biomass can be analyzed for carbon content by conventional TOC measurements and elemental composition can be measured in ash following incineration. The ratio of elements in the biomass provides an estimate of the ratio of elements in the feed water needed to produce this biomass. This approach however, does not, (i) account for carbon which is utilized by the cell, but not incorporated into biomass, or (ii) account for variation in nutrient composition and demands between different cell types in a diverse population. During aerobic degradation in activated sludge systems only between 0.3 and 0.5 units of cell carbon are produced per unit of substrate carbon in high-load conditions, as the remaining part is oxidized to carbon dioxide during cellular respiration (Gallert and Winter 2005). The elemental composition of different bacterial species have been observed to vary by a factor of almost one hundred (Rouf, 1964). While additional requirements for carbon in energy production indicate that biomass composition would overestimate requirements for other nutrients (the true yield of carbon must be less than one), the variability observed between the composition of different bacterial species makes it difficult to conclusively estimate the nutrient requirements for a given system.

Comparing the nutrient to carbon ratios for essential nutrients observed in a greywater, to the same ratios observed in the elemental composition of cell biomass provides an estimate as to the nutrient-balance or limitation of that greywater. Egli (2009) reports an average nitrogen to carbon ratio in generic bacterial cell biomass of 0.24 mg_N_/mg_C_. The nitrogen to carbon ratios reported in Table 1 for four measured hand washing water compositions do not meet this requirement, indicating that hand washing water may be deficient in nitrogen, and warrants investigation for other nutrients as well.

### 1.3 Biologically activated membrane bioreactor

One promising biological treatment system that has demonstrated strong performance recycling wastewater, chemically similar to greywater, is called a biologically activated membrane bioreactor (BAMBi). BAMBi systems feature an ultrafiltration membrane module immersed in a tank of the water to be treated. A biofilm develops on the surface of this membrane that then consumes contaminants from the water as it passes through the biofilm and through the membrane. The permeate water is pumped to a storage reservoir to be reused. Post-treatment systems such as granular activated carbon or chlorine production through electrolysis may be necessary to combat the growth of bacteria in the water during storage (Nguyen et al., 2017).

Unlike conventional membrane bioreactor (MBR) systems, this membrane system does not require any cleaning over the course of standard operation and does not require high-pressure to drive water though the system. BAMBi systems are operated in a gravity-driven membrane (GDM) configuration, resulting in stable flux, without backwashing or other fouling controls (Chomiak et al., 2015; Peter-Varbanets et al., 2010, 2011). The only pressure needed in a GDM system is supplied by the head of the water. Treatment in a BAMBi is predominantly biological, and therefore also requires a balance of nutrients. Household-scale BAMBi systems have demonstrated 95% removal of organic carbon from a wastewater similar in organic loading to hand washing water, but contaminated with feces and urine (Künzle et al., 2015; Ravndal et al., 2015). The contamination of feces and urine in this wastewater (Ravndal et al., 2015) were sufficient to provide a nutrient-balance with respect to carbon. The specific nutrient requirements of a BAMBi system have not been previously investigated.

### 1.4 Goals of this study

The first goal of this study is to understand the quantities and compositions of different inputs to hand washing greywater and how these compositions relate to nutrient-balancing requirements of biological treatment. This analysis must address both the biological compatibility of the carbon and also the macro- and micro-nutrient content of each contributing input and construct a strategy to supplement deficient nutrients.

The second goal is to understand the impact of nutrient-balancing on mechanisms of carbon-removal, and also to evaluate long-term performance and stability of a BAMBi system treating a synthetic hand washing water with nutrient-supplement.

## 2 Material and methods

### 2.1 Batch testing

Batch testing consisted of preparing separate 500 mL volumes of representative and nutrient-balanced synthetic hand washing waters in sterile 1 L Erlenmeyer flasks. The representative hand washing water was designed to recreate a generic hand washing water composition. The nutrient-balanced hand washing water recreated the representative hand washing water composition, but then supplemented the nutrients in which we estimate it to be deficient for biological treatment. The specific composition of the two hand washing water solutions result from the analysis presented in Section 3.1. and are described in detail within that section. The recipe of laboratory ingredients that create each solution are presented in Supporting Information Section S.3. For batch testing, each recipe was diluted to a concentration of 10 mg_C_/L DOC, to reduce inhibitory effects of environmental shock on the bacteria and other effects from having too much surfactant. The chloride concentration was maintained though, following the dilution, with NaCl to 100 mg_Cl_/L. Each flask was then inoculated with 10 mL of bacterial inoculum (Supporting Information Section S.4), incubated at 30 °C and mixed at 120 rpm in the dark, with monitoring of DOC, AOC, growth potential (GP) and total cell count (TCC).

### 2.2 Full-scale testing in BAMBi

**Fig. 1:**
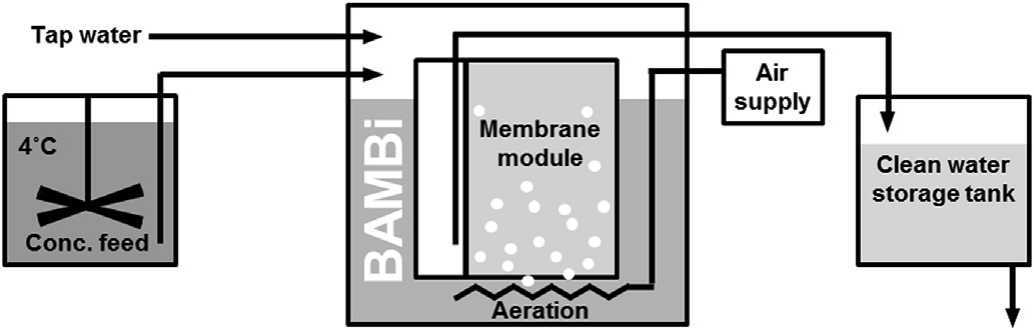
Process schematic for the biologically activated membrane bioreactor (BAMBi) configured as a once-through treatment system.

The BAMBi system consisted of a 58 cm tall standing sandwich membrane module (Microclear MCXL, Newterra, Ontario, Canada) featuring a 150 kDa polyethersulfone ultrafiltration membrane (Microdyn-Nadir, Wiesbaden, Germany) placed into a 52 L wastewater linear low density polyethylene tank (Fig. 1). The BAMBi was fed 75 L/day of nutrient-balanced synthetic hand washing water, in 50 feedings evenly distributed throughout the day. Each feeding event consisted of pumping directly into the BAMBi a 20X concentrated feed (SP Quick Peristaltic pump head and 5001 pumpdrive with Pharmed tubing, Heidolph Instruments, Schwabach, Germany) and an appropriate dilution of non-chlorinated tap water (Tauchpumpe Typ 04, Barwig Wasserversorgung, Bad Karlshafen, Germany). The concentrated feed was stored at 4 °C and mixed at 10 rpm. The general recipe for the feed is presented in Supporting Information Section S.3, however, for full-scale testing tap water was used instead of deionized water. This resulted in some nutrients needing to be added in lesser part, or not at all, to match the same target minimum concentrations. Aeration was provided to the BAMBi at a rate of 20 L/min directly below the membrane module through aeration tubing (Air Curtain 90 cm, Guangdong Risheng, Shenzhen China) producing bubbles approximately 3–4 mm in diameter. Water that passed through the biofilm and through the membrane was collected in a permeate reservoir and then pumped (Tauchpumpe Typ 04) from the reservoir to a clean water storage tank every 5 min. The water level in the BAMBi was maintained at approximately 42 L, which corresponded to approximately 75% of the membrane surface area being submerged in the water. This water level also corresponded with a maximum hydraulic pressure at the bottom of the membrane of approximately 40 mbar. Feeding and permeate pumps were activated using process control hardware (Endress + Hauser AG, Reinach BL, Switzerland), and automation software (Codesys, 3S-Smart Software Solutions GmbH, Kempten, Germany and CitectSCADA, Schneider Electric, Rueil-Malmaison, France).

This BAMBi was initially inoculated with 0.5 L of activated sludge from a municipal wastewater treatment plant. After approximately 6 weeks of operation, steady state performance was achieved. The system was then operated for a period of 100 days, and DOC, ammonium and nitrate were measured. The BAMBi system is intended to completely recycle water in future testing; however, this initial testing was conducted in a once-through configuration permitting greater control of the nutrient levels in the system.

### 2.3 Chemical measurements

DOC was measured using a total organic carbon analyzer (Shimadzu TOC-L, Kyoto, Japan). The sum of ammonium and ammonia was measured with gas-diffusion flow injection (Foss, Hillerød, Demark). Nitrite, nitrate and chloride were measured by ion chromatography (Metrohm 881, Herisau, Switzerland). Nitrogen and sulfur concentrations in soap, humic acid and personal care products were measured with a EuroEA3000 elemental analyzer (Eurovector, Pavia, Italy). Calcium, potassium, magnesium and phosphorus were measured using inductively coupled plasma optical emission spectrometry (Spectro Arcos, Kleve, Germany). Concentrations of all other elements were measured using inductively coupled plasma mass spectrometry (t7500ce ICP-MS, Agilent, Santa Clara, USA).

AOC and GP measurement procedures were based on the work of Hammes and Egli (2005) and Prest et al. (2016). All details of AOC and GP procedures are described in great detail in Supporting Information Section S.5, however AOC and GP both measure the amount of bacterial growth that can be supported by the carbon and other nutrients in the water. In the case of AOC the water sample is spiked with nutrients to ensure carbon limitation. In GP testing, growth is based on nutrient limitation within the sample.

## 3 Results and discussion

The results and discussion section is organized into two main parts. The first part (Sections 3.1) quantifies the mass inputs to hand washing water, and assesses each input for nutrient-balance with respect to biological treatment requirements, and presents a supplementing strategy to correct nutrient-imbalance. The second part (3.2, 3.3) investigates first the differences in mechanism and performance between biological treatment with the realistic (nutrient-deficient) and nutrient-balanced synthetic hand washing waters in batch testing. Then, the long-term performance of the nutrient-balanced solution was investigated in a full-scale BAMBi system.

### 3.1 Quantifying and characterizing inputs to hand washing water and synthetic recipes

The main identified inputs into hand washing water were soap, dirt, dried skin and moisturizer. Representative volumes of liquid soap and washing water per hand washing event have been measured as 1.5 mL soap and 1 L water (Larson et al., 1987). Consumer-oriented hand soaps do not often include a list of ingredients and their concentrations. To overcome this uncertainty in the composition of commercial soaps, a custom liquid soap recipe was developed using exclusively biodegradable ingredients. The components and their relative contributions were selected based on compatible ingredients we do observe in commercial soap products, detailed representative formulations for soap products presented by Hargreaves (2003) and the synthetic greywater composition constructed by Hourlier et al. (2010) and other existing synthetic greywater recipes (presented in Section 1.1.2.). Our custom Eawag soap recipe utilized sodium dodecyl sulfate (SDS, CH_3_(CH_2_)_11_SO_4_Na) as the sole surfactant, Glycerol (C_3_H_8_O_3_) as a moisturizer, 1% sodium chloride (NaCl) as a stabilizing agent and lactic acid (CH_3_CH(OH)COOH) to reduce the pH to approximately 6.5. The final soap recipe was 140 g/L SDS, 50 g/L glycerol, 10 g/L NaCl and 0.7 g/L lactic acid. This soap was then included in the synthetic hand washing water at a ratio of 1.5 mL/L (Larson et al., 1987). The processes for estimating the mass contributions from dirt, skin and moisturizer are based on measurements in the literature and estimation, explained in the Supporting Information Section S.6.

Table 3 summarizes the mass concentrations of all the inputs to hand washing water. By evaluating the relative mass contributions of different hand washing water inputs, we see that soap is tremendously dominant, representing over 90% of the dry mass of inputs to the water. Looking then at individual components of the soap, SDS and glycerol are the most significant ingredients (respectively 69.8% and 24.9% of the total soap dry mass, Table 4), and thus also dominant in hand washing greywater.

**Table 3:** Mass concentrations and relative contributions of inputs to hand washing water.

HWW input	Mass (mg/L)	Water content (%)	Dry mass (mg/L)	Relative contribution (%)
Soap	1500	80	300	90
Dirt	26	0	26	8
Skin	3	20	2.5	1
Moisturizer	8	70	2.5	1
Total	1537	–	331	100

**Table 4:** Mass concentrations and relative contributions of soap components.

Soap component	Dry mass (mg/L)	Relative contribution (%)
SDS	140	69.8
Glycerol	50	24.9
Sodium chloride	10	5.0
Lactic acid	0.7	0.3
Total	200.7	100

The TOC, AOC and concentrations of other nutrients present in each individual contributing material were characterized individually and are presented in Table 5. Three commercial soaps are compared with the custom soap developed for this study. Also examined are the composition of dirt and skin, along with commercially available personal care products that are expected to be present in the hand washing water (sunscreen and skin moisturizer) and tap water. While we acknowledge there could be great variation in the composition of dirt to be washed off hands, we are representing dirt in this study with a compiled dust and a commercially harvested humic acid. All values for soaps, sunscreens and humic acid were measured in our laboratory. Values for dust and skin were assembled from literature sources, explained in greater detail in Supporting Information Section S.7. The elemental composition of tap water is presented for the city of Zürich, Switzerland (City of Zürich, 2016), with an AOC value as measured by Hammes et al. (2006). For each input source, the theoretical concentration required of each element to balance the TOC from that input was calculated and compared to the actual concentration of that element. The estimated nutrient requirements to balance a given concentration of carbon was calculated directly from the elemental composition of biomass compiled from (Egli, 2015; Rouf, 1964), as introduced in Section 1.2, and described in greater detail in Supporing Information Section S.8.

**Table 5:**
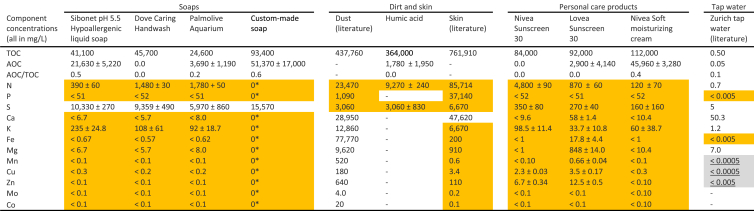
Elemental composition of the inputs to hand washing water (soap, dirt, personal care products and tap water). Shaded cells without underline (orange shade) indicate insufficient content of the element to balance the biological requirements for carbon removal. Shaded cells with underline (grey shade) indicate elements for which the detection limit is higher than the required concentration to balance carbon. A color version of this table is available in the online version, - indicates not measured,* indicates absence based on known ingredients, ± indicates standard deviation, TOC: total organic carbon, AOC: assimilable organic carbon.

The four examined soaps presented large variations in TOC, ranging between 24,600 and 93,400 mg_C_/L. Even greater variation was observed in AOC concentrations. There was no microbial growth at all in one of the commercial soaps, while more than half the organic carbon was assimilated in a second commercial soap and in our custom-made soap. In terms of nutrient-balance, the soaps, as well as all other inputs were determined to contribute insufficient nutrients to balance the carbon. Only sulfur was present in sufficient concentrations to meet the nutrient-requirements in all soaps.

While nitrogen, phosphorus and sulfur contents in dust are insufficient to balance the organic carbon in dust, all other elements exceed the concentrations required for biological nutrient-balance. For the humic acid mixture, in contrast, none of the elements were present in sufficient quantities to balance the carbon. An AOC assay on a 1 mg_C_/L solution of the humic acid mixture yielded a <0.5% reduction in the TOC. Similarly to dust and humic acid, skin contains a high proportion of carbon compared to all other elements. Only calcium is estimated to be present in a concentration sufficient to balance the carbon. The examined sunscreens and moisturizer had similar compositions in terms of carbon (∼100,000 mg/kg) and other nutrients. However, AOC to DOC ratios were tremendously lower in the sunscreens than in the moisturizer, which may be due to the presence of various ingredients toxic to microorganisms like titanium dioxide in the sunscreens. Similarly to the examined soaps, sunscreens and moisturizer were nutrient-deficient with respect to carbon.

Table 6 combines the results from Table 3, Table 5, presenting the elemental contribution of all individual inputs, reflecting the relative mass contributions of each input. Elements present in concentrations below the detection limit were assumed to be completely absent in the hand washing water input. The three most-right columns of Table 6 present the elemental compositions of a representative hand washing water, the estimated biological-nutrient requirements to balance the carbon content of the representative water, and a nutrient-balanced version of the representative water which is supplemented with the nutrients in which we estimate it to be deficient.

**Table 6:**
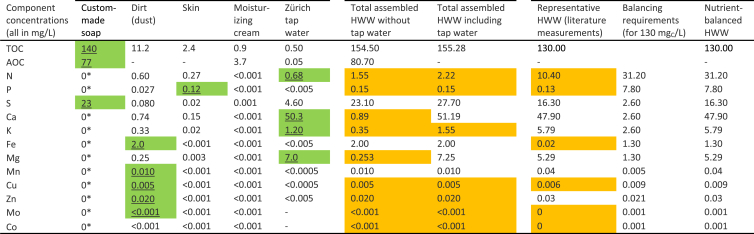
Contributions of the individual inputs to hand washing water (HWW) and composition of representative and nutrient-balanced synthetic formulations. Shaded cells with underline (green shade) indicate the dominant contributing source (including tap water) for each element and shaded boxes without underline (orange shade) indicate nutrient concentrations that are insufficient to balance the carbon in the corresponding hand washing water. - indicates not measured,* indicates absence based on known ingredients. A color version of this table is available in the online version, TOC: total organic carbon, AOC: assimilable organic carbon.

The carbon, nitrogen and phosphorus concentrations in the realistic hand washing water are based on real-world measurements conducted in hand washing water (Jefferson et al., 2004), and the target concentrations for all other elements using measurements conducted in combined greywater (Jefferson et al., 2001). These values are generally consistent with the diversity of measurements presented in Section 1.1.1. The nutrient-balanced composition is based on supplementing any deficient nutrients, to satisfy the nutrient-balancing requirements.

Comparing the “assembled” compositions (based on the sum of identified inputs) to the “representative” composition (based on measured values) demonstrates a general pairwise similarity for the concentration of most nutrients. The assembled and representative compositions also both show (in yellow highlights in Table 6) similar nutrient-deficiencies. We observe each to be deficient in nitrogen and phosphorus and many micro-nutrients. Such deficiencies are expected, though, as we have demonstrated (in Table 5), deficiencies in all contributing inputs. Table 6 also demonstrates the significance of operating a treatment system with recycling or without. The nutrient contribution of the initial water stock could become less significant over time in a recycling system. We estimate substantially lower concentrations of some nutrients (e.g., calcium, magnesium, and potassium) in systems that continuously recycle water, as opposed to systems that treat the water once, and then receive new water with each usage. As tap water is; however, itself not nutrient-balanced, some nutrients must still be supplemented in each case to achieve biological nutrient-balance, however the amounts required could be different. For other nutrients, such as calcium or magnesium, the inclusion of tap water may greatly exceed the biological requirements.

### 3.2 Impacts of nutrient imbalance during short-term experiments

**Fig. 2:**
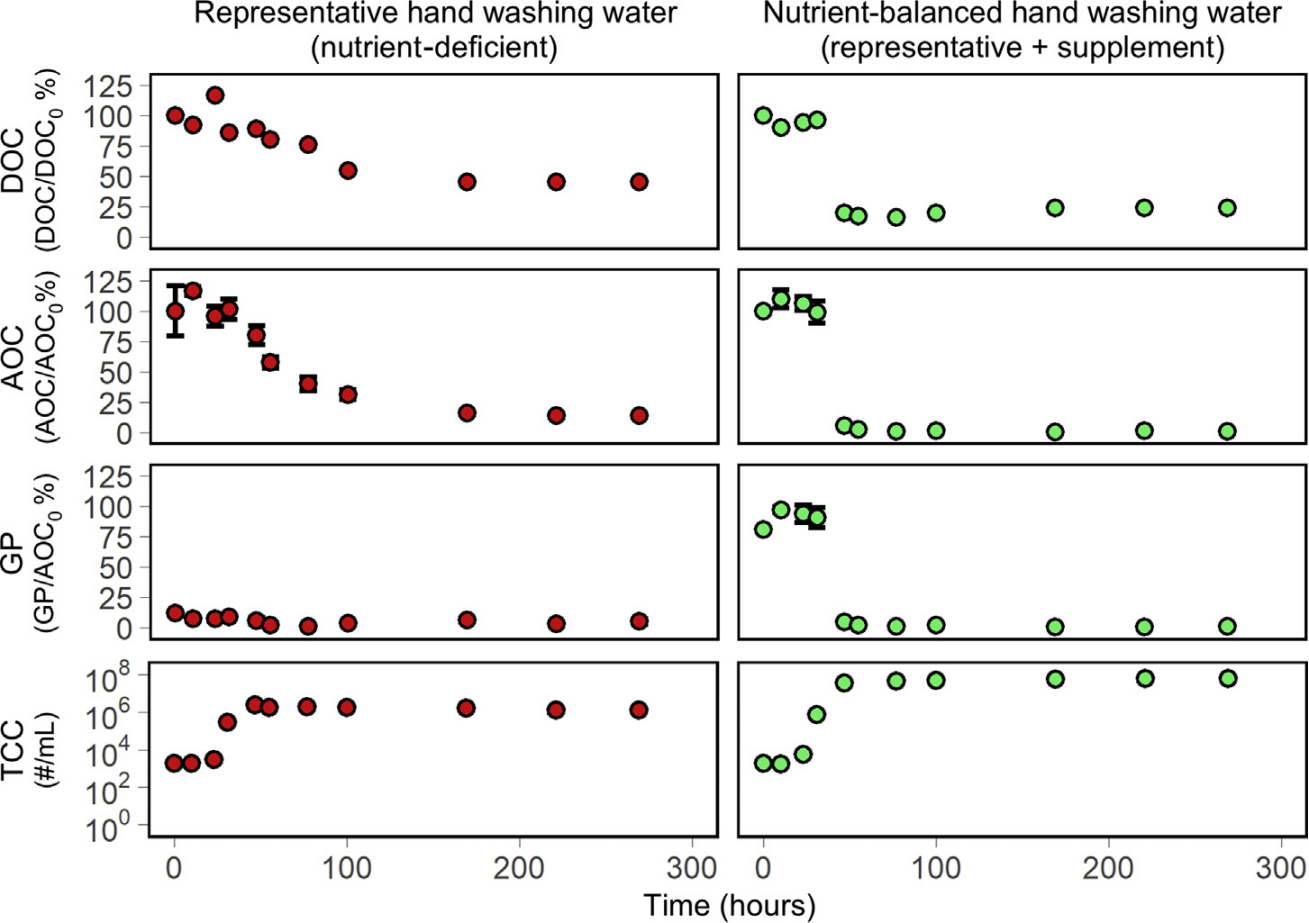
Dissolved organic carbon (DOC), assimilable organic carbon (AOC), growth potential (GP) and total cell count (TCC) during batch testing of a nutrient-deficient representative and a nutrient-balanced synthetic hand washing water. GP is expressed as a percentage of the initial growth observed in the AOC assay.

The impacts of nutrient-balancing were examined in batch testing with one solution formulated to match a representative hand washing water composition and a second solution matching the components of the representative hand washing water, but including additional nutrients, in which we believe the representative solution to be biologically deficient. Each solution was adjusted to contain 10 mg_C_/L TOC to reduce inhibition from excessive surfactant. An inoculum of bacteria was added to flasks of each solution and incubated for 10 days. Changes in the DOC, AOC, GP and TCC values are presented in Fig. 2. To measure AOC, a sample of water is incubated with a bacterial inoculum and spiked with a sufficient concentrations of all other nutrients to ensure that carbon will be limiting. To measure growth potential, in contrast, the sample is incubated without any additional nutrients, and therefore growth can be limited by whatever nutrient is limiting in the sample, not only carbon. Though some bacteria in the inoculum were acclimated to grow on the carbon supplied in the hand washing water, no growth was observed until approximately 24 h of incubation. Following this apparent lag phase, DOC, AOC, growth potential and TCC values for the nutrient-balanced hand washing water all transitioned from initial values to approximately final values within one sample interval (16 h). Both DOC and AOC stabilized at concentrations of approximately 1.7 and 0.09 mg_C_/L respectively (76% and 99% reductions of the initial concentrations). The growth potential in the nutrient-balanced hand washing water followed the same evolution as the AOC, confirming that the nutrient-balanced synthetic hand washing water did contain sufficient concentrations of other nutrients to balance the carbon content of the water. The decreases in organic carbon, AOC and growth potential observed in the batch reactor with nutrient-balancing was accompanied by growth of bacterial cells, finally stabilizing at a TCC of 6.4 × 10^7^ cells/mL.

Testing with the representative hand washing water resulted in a more gradual reduction in organic carbon and a higher final concentration in DOC of 3.5 mg_C_/L (54% reduction) and AOC of 1.2 mg_C_/L (86% reduction). The growth potential of the representative hand washing water was only 15% of the growth potential in the nutrient-balanced hand washing water. This limited growth potential resulted in limited cell growth in the experimental flask, with final concentrations of only 1 × 10^6^ cells/mL. Net cell growth did not continue after 1 day of growth (following 1 day lag); however greater than 80% of AOC remained in the flask, and then 80% of this remaining AOC would be ultimately consumed. Reductions in AOC in the absence of net growth, may be largely attributed to cellular maintenance functions. These two types of processes, growth and maintenance functions have been thoroughly explored for different demands in terms of carbon and energy, but in our context can be considered to be different mechanisms of carbon removal. While the growth-based removal in the nutrient-balanced batch test produced rapid (minutes or hours) reductions in organic carbon, the maintenance-based removal required days (∼6) to reach ultimate removal values.

### 3.3 Long-term testing with nutrient-balanced synthetic hand washing water at full-scale

**Fig. 3:**
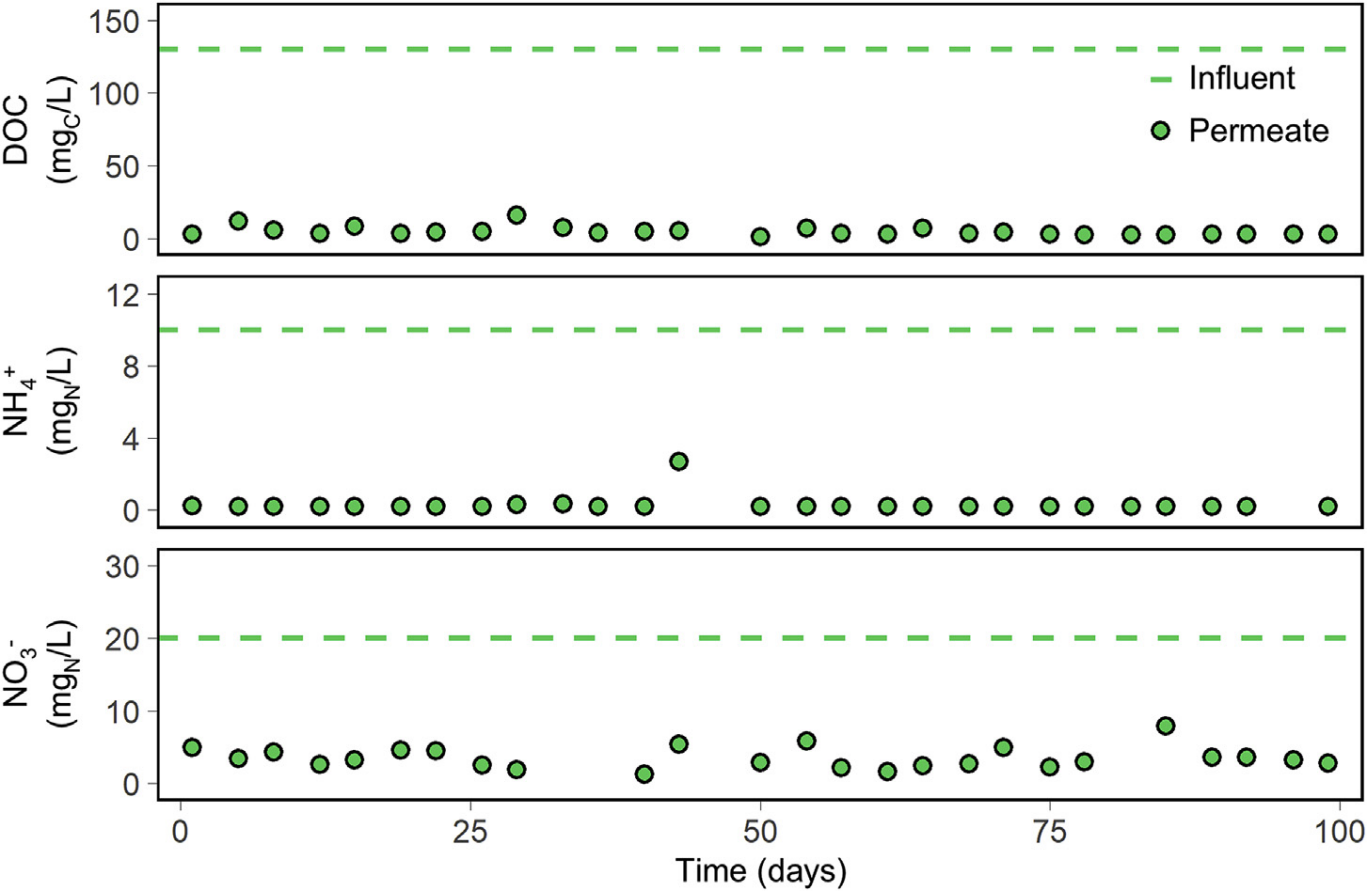
Dissolved organic carbon (DOC), ammonium and nitrate concentration in permeate water of a nutrient-balanced biologically activated membrane bioreactor (BAMBi) treating synthetic hand washing water. Ammonium indicated here is a combination of ammonia and ammonium. Ammonium measurements are reported at a minimum of the detection limit 0.2 mg_N_/L.

Nutrient-balanced synthetic hand washing water was tested in a household-scale BAMBi. Fig. 3 presents concentrations of DOC, ammonium and nitrate in the reactor permeate over 100 days of stable operation. The concentrations of DOC and total nitrogen in the feed water were 130 mg_C_/L and 31 mg_N_/L (10 mg_N_/L of which as ammonium) respectively. The membrane permeate displayed DOC averages of 4.8 ± 3.2 mg_N_/L, ammonium at or close to the quantification level of 0.2 mg_N_/L and nitrate averages of 3.5 ± 1.5 mg_N_/L. A small disruption occurred on day 43, where the ammonium concentration increased to 2.7 mg_N_/L. This was caused by a temporary interruption in the aeration which limited ammonium conversion, but aeration was restored before carbon concentrations in the permeate were significantly impacted. Ammonium concentrations were again below detection (0.2 mg_N_/L) at the following sample point. The ammonium concentration in the permeate was more sensitive to this loss of aeration than the carbon in the permeate (which was not clearly impacted). This behavior may indicate that the normally low ammonium concentration we observe in the permeate may also be dependent on nitrification, and this nitrification was significantly limited while the aeration was off. Our nutrient-balancing strategy may be overestimating nitrogen demands of the system, if our nitrogen supplement can support both nitrification processes and effective carbon removal through cell growth and maintenance. With ammonium generally removed, we expect the ultimate fate of nitrogen that enters the system to be either (i) integrated into biomass through growth, (ii) removed through denitrification, or (iii) reflected in the ∼3.5 mg_N_/L nitrate that exits BAMBi in the permeate. Additional testing could be conducted to better understand how much nitrogen is incorporated into biomass, and how much is removed through denitrification. The presence of nitrate in the permeate also supports the possibility that we are adding more nitrogen than the system truly requires. What our results do clearly show, is that our system is able to achieve 95% removal of the carbon and 90% removal of the nitrogen we do add, which is an strong position from which to explore further optimization.

While we believe that, as in the batch test, nutrient-balancing may also promote cell growth in the BAMBi system, any growth and accumulation of biomass that occurred during our test did not negatively impact the performance in terms of water quality or reduction in membrane flux. Constructive comparisons can be drawn between these residence times within the BAMBi, and the residence times required for carbon removal in the batch tests. The residence time in the full-scale BAMBi is only ∼11 h and the residence time within the biofilm may be only 10–15 min. The shorter timescale (<16 h) for treatment within the nutrient-balanced batch experiment is likely more reflective of the ∼11 h residence time within the BAMBi. In the nutrient-balanced batch test, we observed growth accompanying the carbon removal to a much greater extent than in the batch test without the nutrient-balancing. If the impacts of nutrient-balancing in the full-scale BAMBi system are similar to what we see in the batch tests, we might also expect significant cell growth to occur in the BAMBI, and we might expect that the rate of carbon removal in the BAMBi might be significantly reduced if the system were operated without nutrient-balancing.

### 3.4 Nutrient-balancing in practice

While we have presented variation in literature values for greywater compositions of light greywater, measured in different parts of the world (Section 1.1.1), we have also identified that the dominant inputs to hand washing water are themselves nutrient-deficient (Section 3.1). Therefore, it is likely that despite any variation in the composition of hand washing water, nutrient-deficiency may be omnipresent. Nutrient-supplementing can correct nutrient-imbalance; however, the value of this approach is dependent on, (i) the composition and degree of imbalance in of the water, (ii) the mechanisms of biological carbon removal in the treatment system, and (iii) the required performance in terms of removal.

The representative hand washing water we assembled without nutrient-supplement still contained ∼10 mg_N_/L and sufficient GP to produce 10^6^ cells/mL. Therefore, some organic carbon was removed through a growth-based mechanism and some was removed through a cellular maintenance-based mechanism. Supplementing the realistic composition with nutrients shifts the relative carbon removal system towards growth, and achieves more carbon removal in less time. Requirements of removal rate and ultimate removal to be achieved are application-driven. Full-scale BAMBi reactors in previous studies, treating a similar nutrient-balanced wastewater, achieved 95% carbon removal (Künzle et al., 2015; Ravndal et al., 2015), and produced water that was suitable for hand washing after limited anti-microbial post-treatment (Nguyen et al., 2017). It is possible though that water treated by a biological system without nutrient-balancing might produce a water quality that is acceptable for less-demanding applications, such as toilet flushing, or more-intensive post-treatment may enable usage for more-demanding applications. While post-treatment can improve water quality, optimizing a treatment system to increase the treatment rate for maintenance-based removal may be challenging.

Effective strategies for nutrient-balancing must identify which nutrients to add, how much, but also when to supplement. Estimating nutrient requirements with respect to carbon from generic ash content likely overestimates the true demand. The BAMBi in this study was operated as a once-through system, however accurate nutrient-balancing is likely more critical in a recycling system, where excess nutrients could accumulate over time. Promoting growth through nutrient-balancing may also reduce the start-up time of biofilm systems such as BAMBi, or to help maintain effluent quality during periods of extra waste loading. This study has focused exclusively on hand washing water, however to expand the application of the BAMBi or other biological greywater treatment systems, we would need to understand the nutrient content of these inputs and the biological compatibility of any additional carbon loading. Including water from a kitchen sink may contribute a significant amount of nutrients into the system, however, soap (which can be controlled by the users) may no longer be the dominant input into water from a kitchen sink. Treating greywater from a kitchen sink may also introduce tremendous variability in loading between different locations and at the same location over time.

Part of what makes designing a treatment system exclusively for hand washing water a bit less complex than a system that treats all the greywater from a house involves minimizing variability. We have estimated in this study that soap is the dominate (90% of dry loading) input to hand washing water. When we consider a real world application of our BAMBi system with nutrient-balancing, the soap would also dominate the loading on the system. If the dosage of supplemental nutrients is linked to the usage of the soap, possibly by supplementing nutrients directly into the soap itself, then the nutrient-balance we prescribe for the system can be generally conserved. We suggest then that the performance we achieve in our full-scale BAMBi testing with artificial greywater is likely reflective of performance with real greywater, because the impact of the materials actually washed off the hands is much less significant than the impacts of the inputs that can be controlled (soap and nutrient-supplement).

Variability in loading, uncertainty in compositions of inputs, environmental conditions and many other influences can all impact our ability to realize a treatment strategy in the real world. However we must also consider that each of these influences and our ability to engineer solutions through process design (including nutrient-balancing) may ultimately require different strategies in different systems. Further research is required to better understand the link between the balance of nutrients and the performance of a biological system, specifically in the context of promoting either a growth or a maintenance-based conversion of carbon. This work, however, demonstrates that these pursuits can be rewarding and that understanding when to supplement and perhaps when not to, can have tremendous impacts on the performance of biological systems treating wastewater with nutrient-imbalances.

## 4 Conclusions

• Hand washing soap is the dominant ingredient in hand washing water, accounting for 90% dry mass loading.
• Both hand washing soap and the resultant hand washing greywater are generally estimated to be biologically deficient in terms of nitrogen, phosphorus and other nutrients relative to carbon.
• Supplementing nutrients into hand washing water to match estimated biological nutrient requirements increases the removal rates of organic carbon (and specifically AOC) and achieves lower final organic carbon and AOC concentrations in batch testing with suspended culture, but also promotes cell growth.
• Long-term operation (100 days) of a BAMBi system fed nutrient-balanced hand washing water can deliver effective carbon removal (95%) without detrimental fouling from excessive cell growth or other disruptions.
