# Supplementary material for: Chemical composition, nutrient-balancing and biological treatment of hand washing greywater
Source: Water Res. 2018 Nov 1;144:752–62. doi: 10.1016/j.watres.2018.07.005 (PMC6176911; doi:10.1016/j.watres.2018.07.005)
Supplement: Supporting Information [file mmc1.docx]

Chemical composition, synthetic reproduction, nutrient-balancing and biological treatment of hand washing greywater

Supporting Information

Christopher Ziemba ^a, b^, Odile Larivé ^a, c^, Eva Reynaert ^a, b^, and Eberhard Morgenroth ^a, b, *^

^a^ Eawag: Swiss Federal Institute of Aquatic Science and Technology, 8600 Dübendorf, Switzerland

^b^ ETH Zürich, Institute of Environmental Engineering, 8093 Zürich, Switzerland

^c^ (current address) EPFL Lausanne, Environmental Chemistry Laboratory, 1015 Lausanne, Switzerland

*Corresponding Author. *Email address:* Eberhard.Morgenroth@eawag.ch

Submitted to Water Research on May 13, 2018

Revision Submitted on July 7, 2018

**Section S.1.** Hand washing water composition

This section describes greater background information for the literature studies utilized in the main text.

Laak (1974) determined pollution loads and water quantities used by different types of plumbing fixtures (kitchen sink, bathtub, bathroom sink, laundry machine, water closet) in five households in the USA. The wastewaters were sampled immediately after dishwashing, bathing, laundering, hand or face washing and tooth brushing. The compositions of the wastewaters were reported as biological oxygen demand (BOD), chemical oxygen demand (COD), nitrogen compounds and inorganic phosphate of the water sources. The study is, however, based on samples taken from only five households and it may well be that the mean and the range of values are not representative of the entire US population.

Surendran and Wheatley (1998) measured grab samples originating from the bath or shower, wash basin, washing machine and kitchen sink in a university halls of residence in the UK. A wide range of chemical (BOD, COD, organic and inorganic carbon, nitrogen species, phosphate, copper, lead, zinc, cadmium) and physical (total, suspended, dissolved and volatile solids, turbidity, pH) parameters were measured along with total coliforms and fecal coliforms. The chemical composition of the greywater is likely not representative of the entire British population. For instance, water consumption in the university halls was about double the national average, thus affecting the concentrations of all constituents.

Almeida et al. (1999) investigated the quality of wastewater discharges based on data from two households in England. Samples were taken from domestic appliances (bath, kitchen sink, shower, wash basin and washing machine) in 24 h sessions. Samples were collected and analyzed for various chemical (COD, nitrogen species, phosphate) and physical (total suspended, volatile suspended and dissolved fixed solids) parameters. Measurements were compared to values from literature, and phosphate concentrations found to be significantly lower than literature values. These differences were attributed to the reduction of phosphate content in detergents since the 1970s.

Al-Jayyousi (2003) assessed the role of greywater reuse in sustainable water management with a focus on arid regions. The study reports the composition (BOD, COD, ammonium, phosphate, turbidity and coliform) of water originating from the hand basin, but does not specify the source of the data.

Jefferson et al. (2004) characterized different sources of greywater to evaluate the impact on the selection and operation of water reuse technologies. Greywater samples were collected from 102 individuals in the UK made up of a distribution of ages and gender, separately for bath, shower and hand basins. The samples were analyzed for BOD, COD, total carbon and total organic carbon, nitrogen species, phosphate, turbidity, suspended solids and indicators of fecal contamination (E. coli and fecal streptococci). The organic content in greywater was found to be highly variable.

Similarly to Jefferson et al. (2004), Friedler (2004) assessed the quality of various individual greywater streams in order to determine their potential for different on-site treatment and reuse possibilities. The data provided from 150 samples collected from six different appliances (bath, shower, wash basin, kitchen sink, washing machine and dishwasher) during a sampling campaign in Israel and measured 20 parameters, including seven heavy metals. The average composition of the greywater originating from the wash basin was calculated based on 33 samples.

Jamrah et al. (2008) characterized greywater sources in order to assess the potential for reuse in Oman. Greywater samples from shower, laundry, kitchen and bathroom sink were collected bi-weekly during a period of six weeks from different unspecified locations in Muscat. A wide range of physical, chemical and water quality parameter were reported, including measurements for eight heavy metals.

**Section S.2.** Synthetic Greywater Recipies

Hourlier et al. (2010) formulated a recipe representing greywater from the bathroom to compare the performances of several greywater recycling processes. To be reproducible, technical-quality chemical products in the form of sodium dodecyl sulfate (SDS, surfactant), glycerol (moisturizer), cellulose (organic particles) and lactic acid (acid produced by skin) were used to simulate the organic pollution. Septic effluent was added to provide indicators of fecal contamination. The performances of a direct nanofiltration process were then tested on the synthetic and on a real collected greywater to ensure that the synthetic greywater showed a similar behavior as real greywater when recycled.

Nghiem et al. (2006) developed synthetic greywater recipes to examine the effect of variations in greywater constituents on the fouling of submerged ultrafiltration membranes in greywater recycling. The greywater recipe contained kaolin (inorganic particles), cellulose (organic particles), humic acid (dissolved organic matter), sodium chloride and calcium chloride (salts) and sodium hydrogen carbonate (natural buffer).

Jefferson et al. (2001) aimed at identifying deficient macronutrients and trace elements in greywater and dosing the deficient nutrients to improve the biological treatment of the greywater. The synthetic greywater used in the study was based on a recipe used in the UK water industry representing an average real greywater found in the United Kingdom. It consisted of a mixture of commercial products (soap, hair shampoo, sunflower oil), to which tertiary effluent was added to represent fecal contamination. The characteristics of the synthetic greywater were compared to those of real greywater providing from a greywater collection facility in a university residence hall.

Diaper et al. (2008) developed a greywater technology testing protocol. Their synthetic greywater was designed to simulate greywater from laundry and bathroom from an Australian residential housing. The main products were commercial personal care and household products (shampoo, hand wash, laundry, moisturizer, toothpaste, deodorant and vegetable oil). This mixture was supplemented with a range of analytical-grade products (sodium hydrocarbonate, sodium phosphate, boric acid, lactic acid and clay). Secondary effluent was added as an to represent for fecal contamination.

Surendran and Wheatley (1998) developed a prototype biological process intended to treat greywater from large buildings. The synthetic greywater used to test the prototype comprised soap, detergent, starch, yeast extract and cooking oil. Settled sewage was added to provide an appropriate concentration of bacteria.

Finally, Abed and Scholz (2016) aimed at developing reproducible greywater recipes. They assembled a synthetic greywater using 22 analytical-grade chemicals and secondary treatment effluent (detailed composition not presented). The synthetic greywater simulated several parameters of low-load and high-load greywater, including: biochemical oxygen demand, chemical oxygen demand, nitrogen (ammonia and nitrate), phosphorus (phosphate), pH, redox potential, turbidity and total suspended solids. The chemical characteristics were tested over time and compared to those reported for real greywater.

**Section S.3.** Preparation of synthetic hand washing water

Two synthetic hand washing recipes were utilized in this study. Table S.1 presents the general recipes for each, to be assembled in DI water. The representative hand washing water recipe is designed to recreate the composition of actual hand washing water as measured in literature. This representative recipe is nutrient-deficient. The nutrient-balanced hand washing water recipe matches the composition of the representative recipe, but then also contains additional nutrients designed to meet a target values for expected nutrient-balance. The chloride concentration of each recipe has been elevated to ~100 mg_Cl_/L with the addition of NaCl. Less NaCl must be added to the nutrient-balanced recipe because sodium and chloride are both employed as counterions for the supplemental nutrients in the nutrient-balanced recipe. For batch testing, the generic recipes in Table S.1 have been diluted to 10 mg_C_/L TOC, though the chloride concentration has been maintained by adding NaCl. For full-scale testing, tap water was used in place of DI water. Therefore the concentrations of some elements (such as calcium or magnesium) were omitted from the recipe, because sufficient quantities were present in the tap water. The distinction between always essential or mostly/sometimes essential is provided as described by Egli (2009).

**Table S.****1.** Recipes of laboratory chemicals to create “nutrient-deficient representative” and “nutrient-balanced” synthetic hand washing water solutions.

| **Compound** | **Formula** | **Nutrient-deficient representative**  **hand washing water**  **(mg/L)** | **Nutrient-balanced**  **hand washing water**  **(mg/L)** |
| --- | --- | --- | --- |
| **Carbon Sources and Major Inputs** | | | |
| Sodium dodecyl sulfate (SDS) | NaC_12_H_25_SO_4_ | 210 | 210 |
| Glycerol | C_3_H_8_O_3_ | 75 | 75 |
| Humics (approximate formula) | C_9_H_8_Na_2_O_4_ | 2.5 | 2.5 |
| Kaolin | Kaolin | 23 | 23 |
| Lactic acid | C_3_H_6_O_3_ | 1.08 | 1.08 |
| Sodium chloride | NaCl | 36.7 | 34.0 |
| **Class I Nutrients (always essential)** | | | |
| Ammonium chloride | NH_4_Cl | 39.7 | 39.7 |
| Sodium nitrate | NaNO_3_ | - | 126 |
| Disodium hydrogen phosphate | HNa2O_4_P$\cdot$2H_2_O | 0.75 | 44.8 |
| Sodium sulfate | Na_2_SO_4_ | 20.6 | 20.6 |
| Potassium chloride | KCl | 11.0 | 11.0 |
| Magnesium chloride hexahydrate | Cl_2_Mg$\cdot$6H_2_O | 44.3 | 44.3 |
| **Class II Nutrients (mostly/sometimes essential)** | | | |
| Calcium chloride | CaCl_2_ | 17.3 | 17.3 |
| Iron chloride tetrahydrate | Cl_2_Fe$\cdot$4H_2_O | 0.06 | 4.63 |
| Manganese chloride tetrahydrate | Cl_2_Mn$\cdot$4H_2_O | 0.14 | 0.14 |
| Copper chloride dihydrate | CuCl_2_$\cdot$2H_2_O | 0.016 | 0.016 |
| Zinc sulfate heptahydrate | ZnSO_4_$\cdot$7H_2_O | 0.13 | 0.13 |
| Molybdenum chloride | MoCl_5_ | - | 0.0020 |
| Cobalt chloride hexahydrate | Cl_2_Co$\cdot$6H_2_O | - | 0.0028 |

**Section S.4.** Inoculum preparation

The inoculum was prepared from equal numbers of cells of (i) Evian water (Evian-les-Bains, France) (ii) water treated in a BAMBi system fed a flush water from a source separating toilet (iii) water treated by the BAMBi in this study, and (iv) water collected from the Chriesbach stream (Dübendorf, Switzerland). In order to remove soluable AOC, the BAMBi waters and the stream water were twice centrifuge washed (10 minutes at 5000 g) and re-suspended in filtered (0.2 µm polyethersulfone, Pall) Evian water. The TCC of the inoculum was approximately 80 cells/µL.

**Section S.5.** Measuring assimilable organic carbon (AOC) and growth potential (GP)

Samples for AOC or GP testing were collected using 50 mL syringes and immediately filtered with 0.2 µm polyethersulfone filters (Pall, Port Washington, New York, USA), pre-washed with 50 mL of deionized water. The filtrate was collected into 60 mL glass vials, to which 1 mL of a standardized and diverse inoculum of environmental bacteria was added (Supporting Information Section S.4.). Only for the AOC measurement, additional nutrients in the form of a phosphate-nitrogen buffer, an iron solution and a trace element solution were added in order to ensure carbon limitation. The specifics of these additions are also in accordance with established protocol (Prest et al., 2016). The samples were gently mixed and distributed evenly into triplicate 45 mL glass vials. Flow cytometry (CytoFLEX Flow Cytometer, Beckman Coulter, USA) was used to measure TCC after incubation for 3 to 5 days at 30 °C and 120 rpm. TCC was then converted to AOC using the equation 1 µg AOC = growth of 10^7^ cells (Hammes and Egli, 2005). All glassware was muffled at 450˚C for 4.5 hours. The Teflon-lined screw caps were incubated in sodium persulfate solution (100 g/L) at 60 °C for 1 hour and rinsed with deionized water. AOC measurements of soaps and personal care products were conducted on samples diluted to approximately 1 mg_C_/L as DOC. Testing further dilution of the custom Eawag soap demonstrated dilution to 1 mg_C_/L as DOC, was sufficient to avoid inhibitory affects.

**Section S.6.** Estimating mass contributions of dirt, human skin and moisturizer

The amount of dirt introduced to water during hand washing was estimated to be 30.4 mg of dirt per person per hand washing event (Yamamoto et al., 2006). This value was the median mass of dirt washed off the hands of 4-year-old children. As the hands of the adults are larger (about 2.5 times in surface area), but hopefully the hands of adults are cleaner, we estimate the dirt contribution from our average hand washing user be 25% greater than that of the children. We have assumed that dirt can be represented as agriculturally optimal loam texture with a composition of 25% air, 25% water, 5% organic matter and 45% inorganic matter (Brady, 1990). Therefore we modeled each instance of hand washing requiring 1 L of water, and contributing 23 mg (dry) inorganic material and 2.5 mg (dry) organic material (Nghiem et al., 2006). In the synthetic hand washing water, these two components of dirt were added in the form of kaolin for inorganic and humic acid for the organic part.

The concentration of shedded skin in the hand washing water was calculated based on an average measured skin shed rate of 60 mg/hour (Weschler, 2016). Skin shedding from the hands was assumed to be proportional to the surface of the hands (each 0.05 m^2^) compared to the surface of the entire body (1.8 m^2^) (Kaye and Konz, 1986, Verbraecken et al., 2006). Similarly to above, each hand washing instance was estimated to require 1 L of water with 6.2 hand washing sessions per day (Rossi et al., 2009). We assumed that 25 % of all shedded skin on the hands was washed off during hand washing. Outer layer skin has an average water content of 20% (Verdier-Sévrain and Bonté, 2007). Finally, the amount of moisturizer in the hand washing water was calculated from a dose of 1 mg/cm^2^ skin surface area (Schliemann et al., 2012). We estimated that 50% of the people use moisturizer once a day and 10% of that moisturizer is washed off during hand washing. We again assumed 1 L of water per washing session and 6.2 sessions per day. Water content in moisturizer is estimated to be 70% (Hargreaves, 2003).

**Section S.7.** Estimating elemental compositions of dust and human skin

Representative concentrations of TOC and potassium were taken from measurements of house dust presented in Fergusson et al. (1986). Representative concentrations of phosphorus, molybdenum and cobalt were taken from measurements of street dust by de Miguel et al. (De Miguel et al., 1997). Representative concentrations of calcium, iron, magnesium, manganese, copper, aluminum, and zinc were averaged between the studies of Fergusson et al. (1986) and de Miguel et al. (1997). Nitrogen and sulfur concentrations were taken from García et al. (2007), who measured dust collected from houses and vehicle cabins. All data were converted to a dry mass basis using a 2% moisture content consistent with what was measured in Rasmussen et al. (2011).

The composition of skin lost during hand washing was assumed to match the overall composition of the human body. The concentrations of individual elements were taken from the text of Emsley (1998) , and converted to ppm of dry mass, estimating a 70% water content.

**Section S.8.** Nutrient requirements for biological treatment

Nutrient requirements were estimated based the ratio of elements in typical cell biomass for elements identified as always essential or mostly essential (Egli, 2009), as presented in Table S.2. Nutrients were considered to be balanced when the ratio of that element to carbon in the hand washing water matched the ratio provided.

**Table S.2.** Elemental composition of representative microbial biomass. Values for C, N, P, S, Ca, K, Fe and Mg were taken from Egli (2015), while Zn, Cu, Al, Mn, Mo and Co, were taken from Rouf (1964).

| Element | % dry of cell mass | mg_nutrient_/mg_C_ |
| --- | --- | --- |
| C | 50 |  |
| N | 12 | 0.24 |
| P | 3 | 0.06 |
| S | 1 | 0.02 |
| Ca | 1 | 0.02 |
| K | 1 | 0.02 |
| Fe | 0.5 | 0.01 |
| Mg | 0.5 | 0.01 |
| Zn | 0.008 | 0.00016 |
| Cu | 0.00335 | 0.000067 |
| Mn | 0.0021 | 0.000042 |
| Mo | 0.000265 | 0.0000053 |
| Co | 0.000265 | 0.0000053 |

**References:**

Abed, S.N. and Scholz, M., 2016. Chemical simulation of greywater. Environmental Technology (United Kingdom) 37(13), 1631-1646.

Al-Jayyousi, O.R., 2003. Greywater reuse: Towards sustainable water management. Desalination 156(1-3), 181-192.

Almeida, M.C., Butler, D. and Friedler, E., 1999. At-source domestic wastewater quality. Urban Water 1(1), 49-55.

Brady, N.C., 1990. The Nature and Propeties of Soils., Macmillan Publishing Co., New York, New York, USA.

De Miguel, E., Llamas, J.F., Chacón, E., Berg, T., Larssen, S., Røyset, O. and Vadset, M., 1997. Origin and patterns of distribution of trace elements in street dust: Unleaded petrol and urban lead. Atmospheric Environment 31(17), 2733-2740.

Diaper, C., Toifl, M. and Michael, S., 2008. Greywater Technology Testing Protocol, Austalian Commonwealth Scientific and Industrial Research Organisation (CSIRO).

Egli, T., 2009. Nutrition, microbial. In The Desk Encyclopedia of Microbiology. Schaechter, M. (ed), 788-804, Elsevier Academic Press, Cambridge, Massachusetts, USA.

Egli, T., 2015. Microbial growth and physiology: A call for better craftsmanship. Frontiers in Microbiology 6, 287.

Emsley, J., 1998. The Elements, Clarendon Press, Oxford, UK.

Fergusson, J.E., Forbes, E.A., Schroeder, R.J. and Ryan, D.E., 1986. The elemental composition and sources of house dust and street dust. Science of the Total Environment 50, 217-221.

Friedler, E., 2004. Quality of individual domestic greywater streams and its implication for on-site treatment and reuse possibilities. Environmental Technology 25(9), 997-1008.

García, M., Rodríguez, I. and Cela, R., 2007. Optimisation of a matrix solid-phase dispersion method for the determination of organophosphate compounds in dust samples. Analytica Chimica Acta 590(1), 17-25.

Hargreaves, T., 2003. Chemical Formulation: Overview of Surfactant-based Preparations Used of Everyday Life, The Royal Society of Chemistry, Cambridge, UK.

Hourlier, F., Masse, A., Jaouen, P., Lakel, A., Gerente, C., Faur, C. and Le Cloirec, P., 2010. Formulation of synthetic greywater as an evaluation tool for wastewater recycling technologies. Environmental Technology 31(2), 215-223.

Jamrah, A., Al-Futaisi, A., Prathapar, S. and Harrasi, A.A., 2008. Evaluating greywater reuse potential for sustainable water resources management in Oman. Environmental Monitoring and Assessment 137(1-3), 315-327.

Jefferson, B., Burgess, J.E., Pichon, A., Harkness, J. and Judd, S.J., 2001. Nutrient addition to enhance biological treatment of greywater. Water Research 35(11), 2702-2710.

Jefferson, B., Palmer, A., Jeffrey, P., Stuetz, R. and Judd, S., 2004. Grey water characterisation and its impact on the selection and operation of technologies for urban reuse. Water Science and Technology 50, 157-164.

Kaye, R. and Konz, S., 1986. Volume and Surface Area of the Hand. Proceedings of the Human Factors Society Annual Meeting 30(4), 382-384.

Laak, R., 1974. Relative pollution strength of undiluted waste materials discharge in household and the dilution waste used for each. In Manual of Gray Water Treatment Practice. Winneberger, J.H.T. (Ed.), 68-78, Ann Arbor Science, Ann Arbor, Michican, USA.

Nghiem, L.D., Oschmann, N. and Schäfer, A.I., 2006. Fouling in greywater recycling by direct ultrafiltration. Desalination 187(1-3), 283-290.

Prest, E.I., Hammes, F., Kötzsch, S., Van Loosdrecht, M.C.M. and Vrouwenvelder, J.S., 2016. A systematic approach for the assessment of bacterial growth-controlling factors linked to biological stability of drinking water in distribution systems. Water Science and Technology: Water Supply 16(4), 865-880.

Rasmussen, P.E., Beauchemin, S., Chénier, M., Levesque, C., MacLean, L.C.W., Marro, L., Jones-Otazo, H., Petrovic, S., McDonald, L.T. and Gardner, H.D., 2011. Canadian House dust study: Lead bioaccessibility and speciation. Environmental Science and Technology 45(11), 4959-4965.

Rossi, L., Lienert, J. and Larsen, T.A., 2009. Real-life efficiency of urine source separation. Journal of Environmental Management 90(5), 1909-1917.

Rouf, M.A., 1964. Spectrochemical Analysis of Inorganic Elements in Bacteria. Journal of bacteriology 88, 1545-1549.

Schliemann, S., Petri, M. and Elsner, P., 2012. How much skin protection cream is actually applied in the workplace? Determination of dose per skin surface area in nurses. Contact Dermatitis 67(4), 229-233.

Surendran, S. and Wheatley, A.D., 1998. Grey-water reclamation for non-potable re-use. Journal of the Chartered Institution of Water and Environmental Management 12(6), 406-413.

Verbraecken, J., Van De Heyning, P., De Backer, W. and Van Gaal, L., 2006. Body surface area in normal-weight, overweight, and obese adults. A comparison study. Metabolism: Clinical and Experimental 55(4), 515-524.

Verdier-Sévrain, S. and Bonté, F., 2007. Skin hydration: A review on its molecular mechanisms. Journal of Cosmetic Dermatology 6(2), 75-82.

Weschler, C.J., 2016. Roles of the human occupant in indoor chemistry. Indoor Air 26(1), 6-24.

Yamamoto, N., Takahashi, Y., Yoshinaga, J., Tanaka, A. and Shibata, Y., 2006. Size distributions of soil particles adhered to children's hands. Archives of Environmental Contamination and Toxicology 51(2), 157-163.
